# Supplementary material for: ICF1-Syndrome-Associated DNMT3B Mutations Prevent De Novo Methylation at a Subset of Imprinted Loci during iPSC Reprogramming
Source: Biomolecules. 2023 Nov 28;13(12):1717. doi: 10.3390/biom13121717 (PMC10741953; doi:10.3390/biom13121717)
Supplement: Supplementary file 1 [file biomolecules-13-01717-s001.zip › Supplementary files_biomolecules_2566524/Verma_et_al_Supplementary_Figures_biomolecules-2566524_proof.pdf]

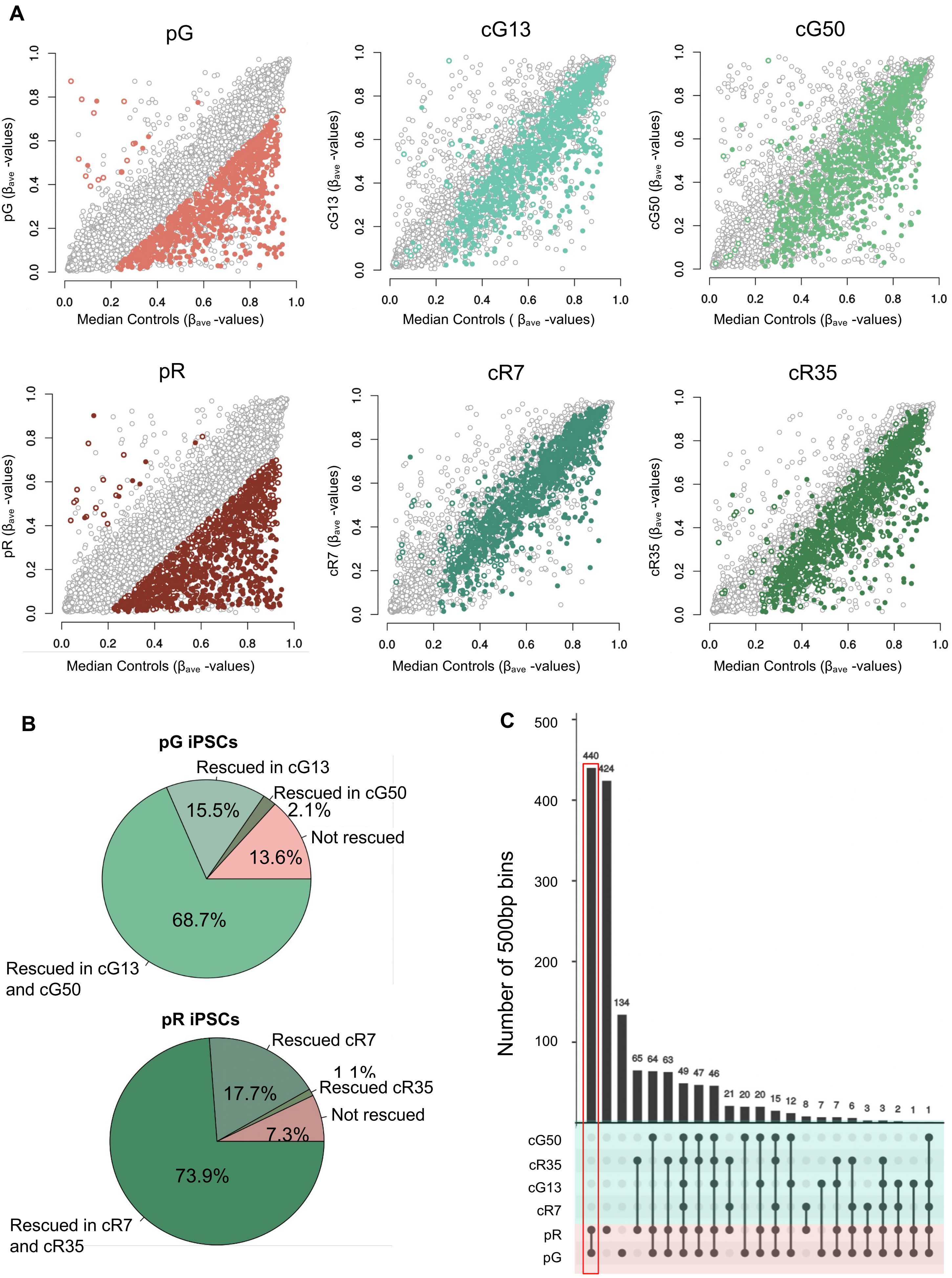

A
pG
cG13
cG50
pR
cR7
cR35
Median Controls (β_{ave} -values)
pG (β_{ave} -values)
cG13 (β_{ave} -values)
cG50 (β_{ave} -values)
pR (β_{ave} -values)
cR7 (β_{ave} -values)
cR35 (β_{ave} -values)
B
pG iPSCs
Rescued in cG13
15.5%
Rescued in cG50
2.1%
Not rescued
13.6%
68.7%
Rescued in cG13 and cG50
pR iPSCs
Rescued cR7
17.7%
1.1%
Rescued cR35
Not rescued
7.3%
73.9%
Rescued in cR7 and cR35
C
Number of 500bp bins
440 424 134 65 64 63 49 47 46 21 20 20 15 12 8 7 7 6 3 3 2 1 1
cG50
cR35
cG13
cR7
pR
pG

Supplementary Figure 1

A, Scatter plots show the methylation profiles of ICF1 iPSCs (pG and pR) and respective CRISPR/Cas9 corrected iPSCs (cG13, cG50 and cR7 and cR35) (y-axis) compared to median β_{ave}-value of controls iPSCs (n=11) (x-axis). Each dot corresponds to a 500bp bin (n=34,048). Grey dots account for non-differentially methylated 500bp bins. Differentially methylated (dm) 500bp bins are represented in colored dots (pG, n=940; pR, n=1285). The color filled circles (n=767) represent the dm-500bp bins that are shared between both pG and pR, while unfilled circles are affected in either pG (n=173) or pR (n=518) iPSCs. **B**, Pie charts represent the proportion of differentially methylated 500bp bins (dm-500bp bins) in ICF1 iPSCs (pG, n=940, top panel; pR, n=1285, bottom panel) and ICF1 corrected 500bp bins in cG13/cG50 and cR7/cR35, respectively. **C**, Upset plot shows the number of 500bp bins which are differentially methylated in ICF1 iPSCs and their methylation recovery status in cG13/cG50/cR7/cR35. Black and grey dots represent presence or absence of differentially methylated 500bp bins in the corresponding iPSCs, respectively. The red outline highlights the 500bp bins (440) where the methylation defect is present (black dot) in both patient iPSCs and rescued in all corrected iPSCs (grey dots). 424 and 134 500bp bins were differentially methylated specifically in pR and pG, respectively. Black lines identify bins that are affected across the corresponding iPSCs.

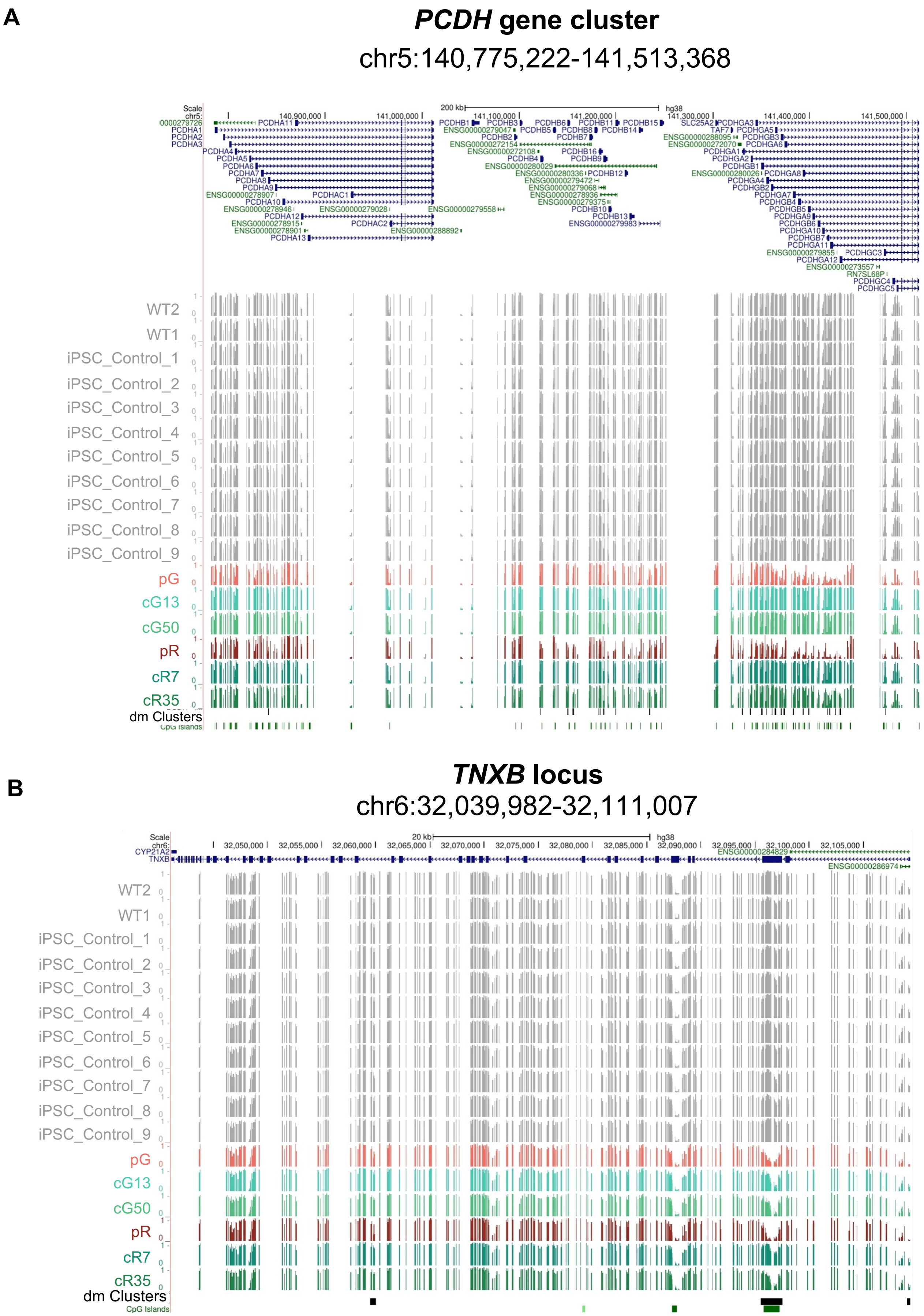
A
PCDH gene cluster
chr5:140,775,222-141,513,368
WT2
WT1
iPSC_Control_1
iPSC_Control_2
iPSC_Control_3
iPSC_Control_4
iPSC_Control_5
iPSC_Control_6
iPSC_Control_7
iPSC_Control_8
iPSC_Control_9
pG
cG13
cG50
pR
cR7
cR35
dm Clusters
B
TNXB locus
chr6:32,039,982-32,111,007
WT2
WT1
iPSC_Control_1
iPSC_Control_2
iPSC_Control_3
iPSC_Control_4
iPSC_Control_5
iPSC_Control_6
iPSC_Control_7
iPSC_Control_8
iPSC_Control_9
pG
cG13
cG50
pR
cR7
cR35
dm Clusters
CpG Islands

Supplementary Figure 2

A, UCSC snapshot depicts the methylation coverage of iPSC controls, ICF1 iPSCs and their corrected clones as β-values at *PCDHA*, *PCDHB* and *PCDHG* gene clusters. The locus is known to lose methylation in ICF1 syndrome [24,27] and to recover normal methylation levels at several CpGs upon DNMT3B correction by CRISPR/Cas9. **B,** UCSC snapshot of *TNXB* gene shows DNA hypomethylation that is only partially restored in corrected iPSC clones. Differentially methylated 500bp bins and CpG Islands are represented by color blocks below the tracks. Differentially methylated (dm) Clusters are shown at the bottom of UCSC tracks.

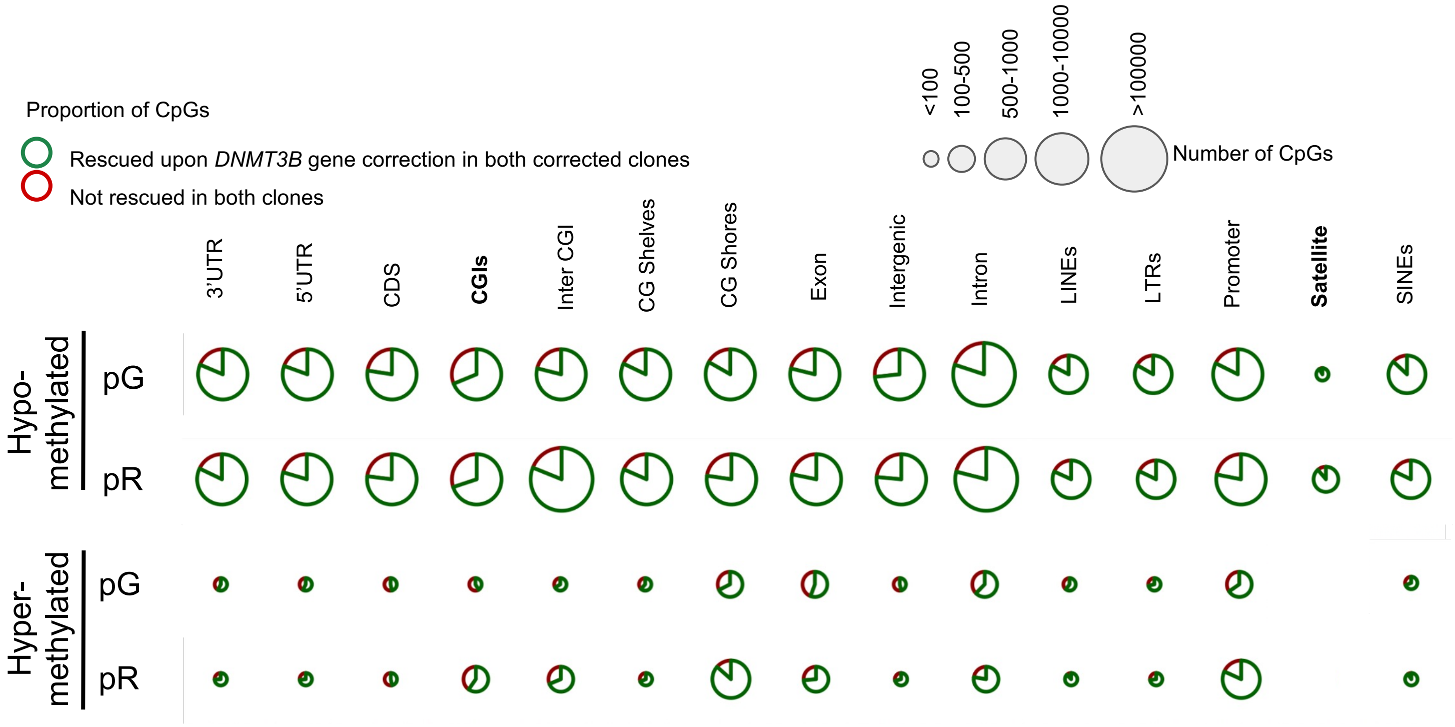

Proportion of CpGs
Rescued upon *DNMT3B* gene correction in both corrected clones
Not rescued in both clones
<100
100-500
500-1000
1000-10000
>100000
Number of CpGs
3'UTR
5'UTR
CDS
CGIs
Inter CGI
CG Shelves
CG Shores
Exon
Intergenic
Intron
LINEs
LTRs
Promoter
Satellite
SINEs
Hypo-methylated
pG
pR
Hyper-methylated
pG
pR

Supplementary Figure 3

Pie charts depict the proportion of differentially methylated CpGs in pG and pR iPSCs annotated to various genomic elements. The outline of the pie chart represents CpGs remaining hypo- or hypermethylated (red arc) following DNMT3B correction in at least one clone, or that are rescued in both corrected clones (green arc). The size of the pie represents the number of CpG analyzed in each of the genomic elements. CGIs and Satellites are in bold as they show the least and the highest percentage of methylation recovery upon correction, respectively. No hypermethylated satellite sequences have been found in ICF1 iPSCs.

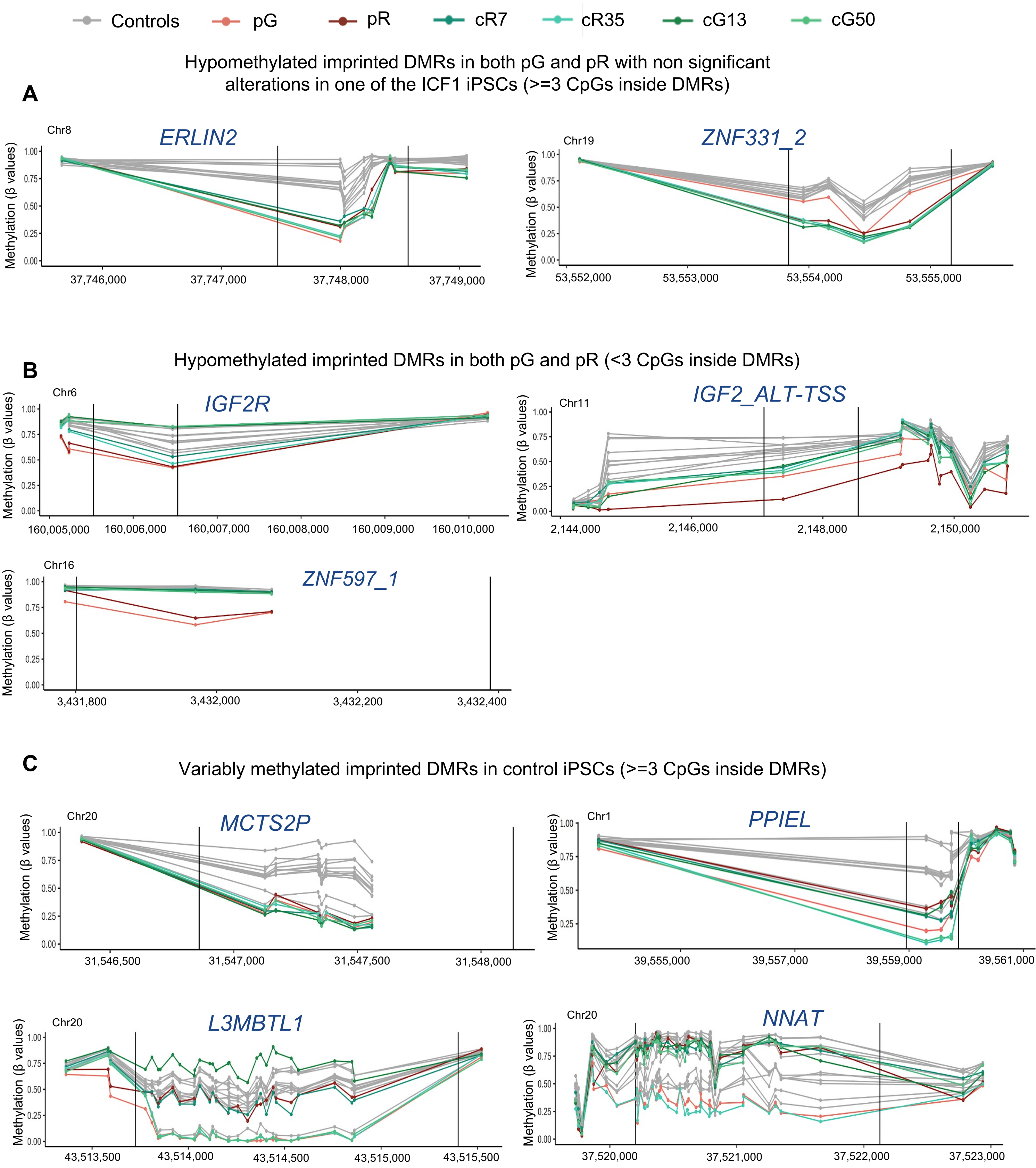

Controls
pG
pR
cR7
cR35
cG13
cG50
A
Hypomethylated imprinted DMRs in both pG and pR with non significant alterations in one of the ICF1 iPSCs (>=3 CpGs inside DMRs)
Chr8
ERLIN2
Methylation (β values)
37,746,000
37,747,000
37,748,000
37,749,000
Chr19
ZNF331_2
53,552,000
53,553,000
53,554,000
53,555,000
B
Hypomethylated imprinted DMRs in both pG and pR (<3 CpGs inside DMRs)
Chr6
IGF2R
160,005,000
160,006,000
160,007,000
160,008,000
160,009,000
160,010,000
Chr11
IGF2_ALT-TSS
2,144,000
2,146,000
2,148,000
2,150,000
Chr16
ZNF597_1
3,431,800
3,432,000
3,432,200
3,432,400
C
Variably methylated imprinted DMRs in control iPSCs (>=3 CpGs inside DMRs)
Chr20
MCTS2P
31,546,500
31,547,000
31,547,500
31,548,000
Chr1
PPIEL
39,555,000
39,557,000
39,559,000
39,561,000
Chr20
L3MBTL1
43,513,500
43,514,000
43,514,500
43,515,000
43,515,500
Chr20
NNAT
37,520,000
37,521,000
37,522,000
37,523,000

Supplementary Figure 4

Line charts depict DNA methylation profiles at the hypomethylated imprinted loci in ICF1 iPSCs, corrected iPSC lines and controls (n=11) shown in Figure 2A. The charts present the imprinted DMRs hypomethylated in both pG and pR (>=3 CpGs inside DMRs) with insignificant alterations in one of the ICF1 iPSCs **(A),** those hypomethylated in both pG and pR but showing less than 3 CpGs inside a DMRs **(B)**, and those variably methylated among control iPSCs (>=3 CpGs inside a DMRs) **(C)**. Dots on the line show the position of the CpGs. Two black lines within the plot represent the span of the iDMR. The x-axis represents genomic coordinates in and around the iDMRs, while the y-axis shows methylation levels (β-values).

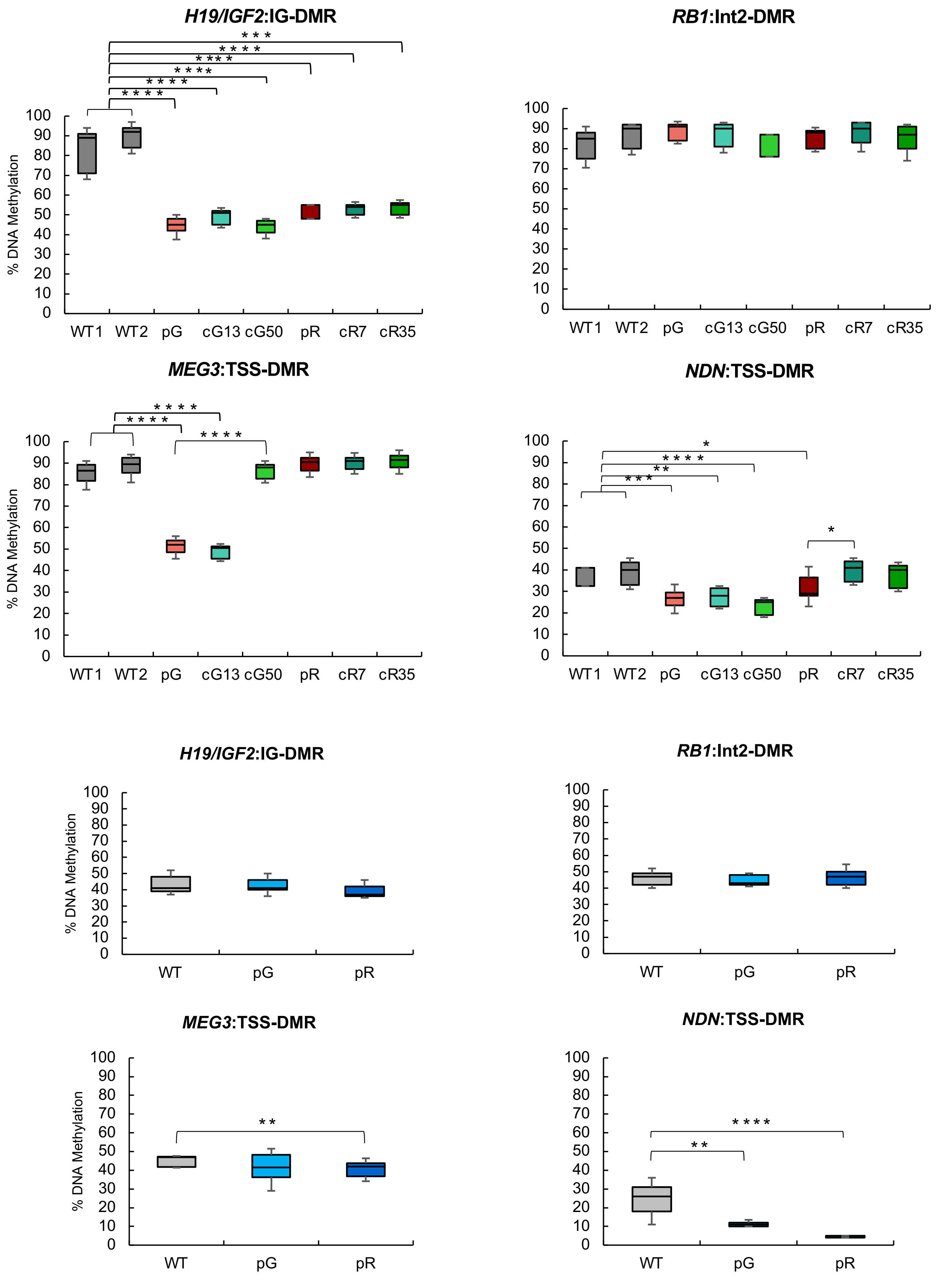

H19/IGF2:IG-DMR
RB1:Int2-DMR
MEG3:TSS-DMR
NDN:TSS-DMR
% DNA Methylation
WT1 WT2 pG cG13 cG50 pR cR7 cR35
H19/IGF2:IG-DMR
RB1:Int2-DMR
MEG3:TSS-DMR
NDN:TSS-DMR
WT pG pR

Supplementary Figure 5

Boxplot showing DNA methylation analysis of four imprinted DMRs in WT, patient and corrected iPSCs (upper panels) and in patient pR and pG parental fibroblasts (lower panels), as measured by bisulfite pyrosequencing. *P* values have been calculated by two-tailed Student's t test (** $p < 0.01$; ***$p < 0.001$; ****$p < 0.0001$).

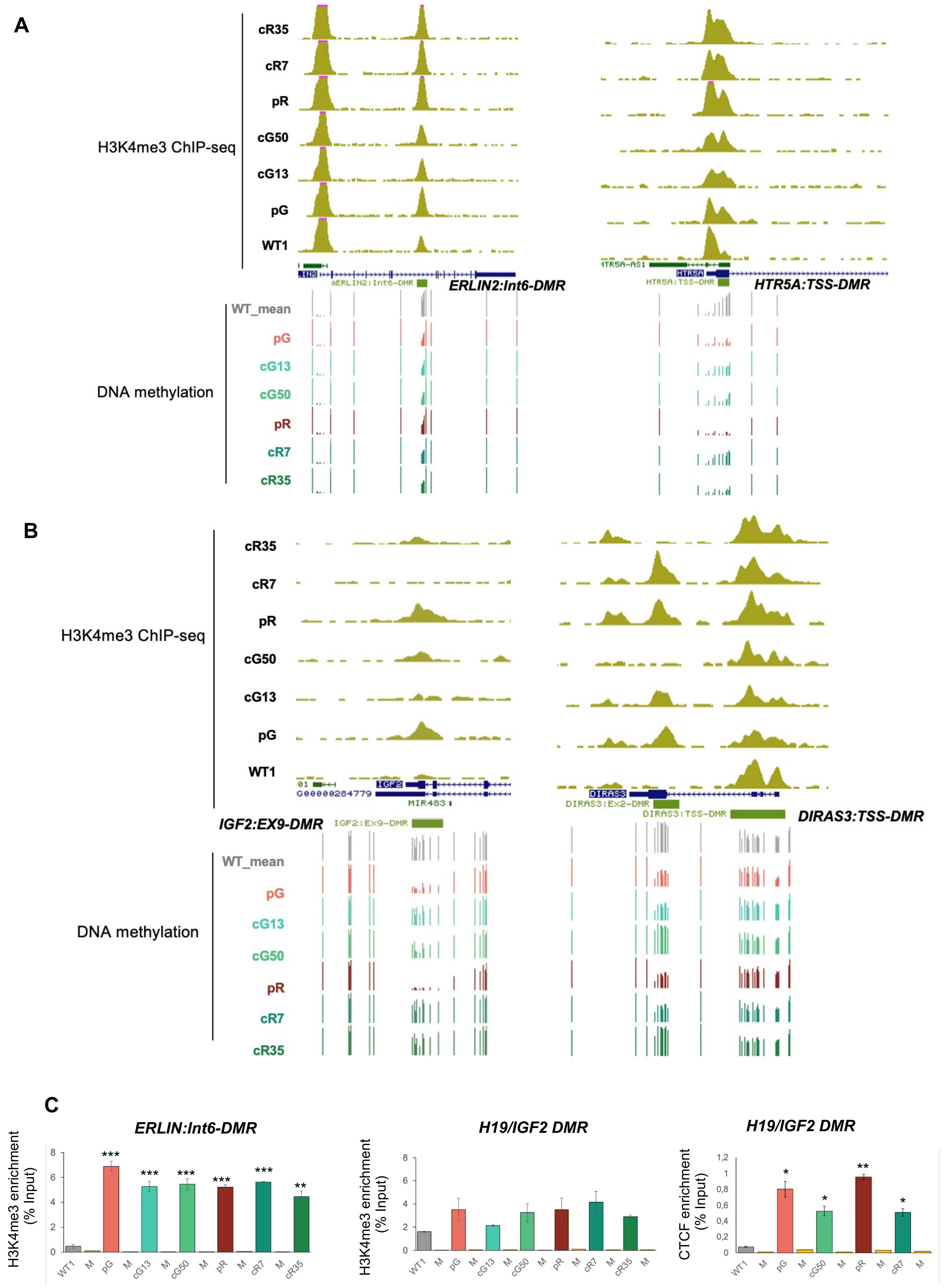
A
H3K4me3 ChIP-seq
cR35
cR7
pR
cG50
cG13
pG
WT1
ERLIN2:Int6-DMR
HTR5A:TSS-DMR
DNA methylation
WT_mean
pG
cG13
cG50
pR
cR7
cR35
B
H3K4me3 ChIP-seq
cR35
cR7
pR
cG50
cG13
pG
WT1
IGF2:EX9-DMR
DIRAS3:TSS-DMR
DNA methylation
WT_mean
pG
cG13
cG50
pR
cR7
cR35
C
ERLIN:Int6-DMR
H3K4me3 enrichment (% Input)
WT1 M pG M cG13 M cG50 M pR M cR7 M cR35
H19/IGF2 DMR
H3K4me3 enrichment (% Input)
WT1 M pG M cG13 M cG50 M pR M cR7 M cR35 M
H19/IGF2 DMR
CTCF enrichment (% Input)
WT1 M pG M cG50 M pR M cR7 M

Supplementary Figure 6

Genome browser view of the *IGF2:Ex9* and *DIRAS3:Ex2* **(A)** and *HTR5A* and *ERLIN2* **(B)** imprinted DMRs (green bars below the GENCODE V43 gene tracks). The coverage tracks from the top to the bottom display the H3K4me3 enrichment levels (light green) obtained by previously published ChIP-Seq data [24] and the methylation level shown as β-values for all iPSCs (control, ICF1, and corrected iPSC clones) obtained in this study.
C, ChIP-qPCR measuring H3K4me3 levels at *ERLIN:Int6-DMR* and *H19/IGF2 DMR* (left and middle panels) and CTCF binding level (right panel) in WT, ICF1 and corrected iPSCs. Amplicon enrichment in immunoprecipitated and mock samples (M, orange bars) is expressed as percentage (%) of input. Bars and error bars represent means and SEM of at least three experimental repeats. Statistical analysis was performed using a one-tail two-sample Student's t-test compared to WT1 (*$p < 0.05$, **$p < 0.01$, ***$p < 0.001$).
